# Some Practical Facts Relating to the Trance State in Inebriety

**Published:** 1883-04-20

**Authors:** T. D. Crothers


### SOME PRACTICAL FACTS RELATING TO THE TRANCE STATE IN INEBRIETY.

By T. D. Crothers, M. D.

In 1877 my attention was called to an inebriate who while sober had purchased a trotting horse, paying a fabulous price, giving a note of hand in part payment. Two days after, he denied all knowledge of the transaction, and became involved in a legal contest. On the trial it appeared that the purchase of the horse had been discussed for a day or more, and that he had exhibited unusual sagacity and judgment to avoid deception and protect himself. Also that, although drinking large quantities of spirits, he gave no evidence of other than good judgment, and perfect knowledge of his acts and their consequences.

In the defense it was shown that the purchase of the horse was a most unusual act; that he rarely ever visited the race course; was afraid of driving fast horses; never took any interest in racing; had many horses of his own; and lastly, needed the money paid for this horse for another purpose which had been determined before. From his own statement he had many blanks of memory while drinking, and at this time he lost all recollection of passing events from the hour of dinner, during which he drank freely, until the second day after, when he awoke in his store, and supposed he had been asleep a short time. His family were sure that in these blank states they could recognize a dullness of mental action and a general abstrcctedness of manner not common when sober. He could not read in this state, and seemed incapable of fixing attention on the conversation for only a few moments at a time.

The suit went against him, and soon after he came to the asylum for treatment. His inebriety grew out of inheritance, overwork, and bad nutrition, and was noted for periods of continuous drinking and free intervals of sobriety, both of which were irregular, and not prominent in any particular.

From inquiry I was surprised to find that these blank states were not uncommon among inebriates; that nearly every case gave a history of loss of memory and consciousness of acts committed while using spirits to excess. In some cases this blank was total and remained so ever after; in others it was partial and cleared up after recovery. Acts committed during the delirium of intoxication, or in mental states approaching this, or stupor, would not be remembered, for some time after, but would gradually be recalled until it was all clear. In other cases this blank of memory would remain like a cloud for weeks, then all at once, from some little circumstance, break away, and every act be perfectly clear.

These freaks of memory, (so called), following excess of alcohol, are almost infinite in variety and complexity, and are very significant when studied carefully. Where the blank is total, and remains so, during which the person acts more or less rationally, and never after is able to recall any recollection of it, it is called a trance state.

In all probability this is a suspension or paralysis of certain brain functions, or as Dr. Beard believes, a consciousness that is not rememberable. In other words, the person in this state acts as if he was in full possession of all his brain powers, and yet the memory does not record it for future consideration. He is for the time being an automaton,—acts and apparently reasons from some unknown stand-point.

Whatever the pathology may be, it is clear that the mind in this state is a mental waif, subject to every passing influence, both from disordered states within and without.

From my experience this state is very common in inebriety, and closely associated with many of the criminal and insane acts committed at this time.

As in all other new phases of science, there is a psychological element, which must be understood before its mysteries can be cleared away. Take any case of a chronic inebriate seen on the streets, and study the operations of his mind and the nature of his actions, while under the influence of alcohol, and this element will be clear.

The inebriate suffering from excess of alcohol has always a disorganized brain power; various governing centres are suspended, mental and physical incoordination is present, and he is literally unsound, insane, and irresponsible.

If the trance state is present, he is a dangerous automaton, moving along certain fixed lines of conduct, or acting in obedience to unknown forces which may change or vary any moment.

Thoughts and impulses suggested by deranged organs, or coming from the past, may suddenly concentrate into action, irrespective of consequences. Both subjective and objective states, influenced by conditions of health and brain power, may develop into deeds that are practically unknown and unrecorded by the higher brain centres.

The strange, unaccountable acts of the inebriates who are not stupid or delirious, have been attributed to vice and low moral conditions. Yet a study of these cases reveals an absence of purpose and motive, and a class of actions that are devoid of all sense and common reason. For instance, a man who was an inebriate, while drinking and apparently conscious, sold out his farm at a very low rate. He had no recollection of this event, and there was no reason or motive for doing it. In one case a man forged a note and drew the money on it, while drinking, without any object or motive. He had no recollection of this, and had money at his command, and no possible use for the other funds.

In another instance a man in this state offered violence to his father and mother, and was thoroughly unconscious of what he had done. These cases can be multiplied to almost an indefinite extent; and beside having no recollection of the act, they are marked by an absence of all reason or purpose, that indicates their real nature. They are not alone confined to cases of inebriety, but are seen following blows on the head and shocks from traumatism, either physical or psychical. The following cases are marked instances:

A leading physician was rendered insensible by an injury from a railroad accident; he recovered, went about assisting the wounded, was finally sent home, and regained consciousness, or apparently woke up, thirty-six hours after, having not the slightest recollection of anything which had happened from the time of the accident, in which he was precipitated to the bottom of the car.

A surgeon in one of the large cities was thrown out of a carriage, striking on his head, and was unconscious for a few moments, got up and went to a hospital, wrote many prescriptions, directing the surgical dressing in several cases, came home and laid down, sleeping and drowsy for twelve hours, then awoke, having no recollection of all the time from the moment of the accident.

In these cases there was a trance state following the injury, although there were no signs of this in the manner or actions of the persons. In inebriety such cases are those in which the mind moves along accustomed lines of thought and action, following some plan which has been usual or common in the past.

Epilepsy, insanity, or the emotions powerfully excited, may be, and often are, followed by these automatic phenomena. Instances of such cases are to be found in all medical literature relating to mental diseases, although not recognized by this name or understood. In inebriety there is a peculiar disposition to develop this form of mental disturbance, and its full recognition will mark an era in the progress of science. It is always found associated with a peculiar neurotic condition, either induced by alcohol or existing before alcohol is used. It is also seen most frequently in chronic cases.

The practical facts which should be noted are not only to determine the presence of inebriety in a given case, but to study the character and nature of the mental state, and the actions which result from it. No one who uses alcohol to excess can be of sound mind. There are always instability and perversion of mental activity, as well as defective brain power.

The higher brain centres governing the relations of life are disorganized, and morbid impulses of any kind are likely to take possession and guide mental activity. No one can draw dividing lines where normal reason is lost in cloudy, unstable action.

When the inebriate, by his manner and conduct, exhibits manifest hallucinations, delirium, and unnatural stupor or excitement, it is not difficult to recognize the mental disturbance. But when his action and manner are along the line of natural, every-day life, although he may be using alcohol to excess, and claims to have no recollection of what he has been doing, the case should receive the closest scrutiny.

When a drinking man, not stupid or delirious from alcohol, but known to be using it all the time, commits an extraordinary or unusual act, not in accordance with any reasonable motive or purpose, and afterwards denies all recollection of what he did, the possibility of a trance state is very strong.

It has been well established that inebriates have, through some motive of their own, or by the persuasion of others, deliberately used alcohol for the purpose of giving them some power to commit crime; also hoping by this means to shield themselves from the legal consequences of their acts.

These cases are marked by either an unreasoning frenzy or a cool deliberation that is foreign to the man in his natural condition. The following case was very prominently discussed a few years ago. A mild, quiet man, who was a continuous drinker, and had never been known to do a violent act, committed a most aggravated murder of a man, who was on the best of terms with him, and for no visible purpose. He denied all knowledge of the act, and died in prison before his execution, from consumption following excessive grief. Some years after it appeared that after drinking a certain amount he was unconscious of the nature of his acts, but could be influenced by any strong will, and had been often persuaded to do certain things that were not natural in his sober moments.

It also came out that this murder was planned and urged by two men, who were fully aware of his condition of oblivion to the nature and consequences of his acts; that by continually plying him with spirits, and urging this act, it became impressed on his cloudy brain, and was carried out. This was a condition of trance, which had been noticed and taken advantage of by the real perpetrators of the crime. I am convinced that much of the purposeless crime committed by inebriates, which puzzles both courts and juries to explain on any reasonable basis of human conduct, originates in the trance state. Such cases are never studied, and are unknown, and the legal efforts to remedy them by punishment literally precipitate them lower, and make recovery more and more difficult.

In an article "On the Trance State in Inebriety," read before the New York Medico-Legal Society, last year, I have detailed some very significant cases, showing the necessity of careful study of all these cases, by competent men, before the nature of responsibility in any case can be determined. The plea of no recollection or memory of the act or event urged by the inebriate is always a physiological and psychological possibility, which can only be settled by a careful study.

So far my studies have indicatad that the trance state in inebriates is marked by two forms of mental action that can be determined clearly; one in which the mind in this state moves along certain familiar lines of action and follow some purpose which has been previously fixed, all of which appears natural and reasonable. The second form is where a new line of thought and action appears, unusual and foreign to his every-day life—often impulsive, inconsistent, and yet seemingly one which he is fully conscious of, and if questioned at the time, may give reasons that seem to justify his conduct.

In both of these forms, sudden changes may occur. Emotional disturbances may precede this state, or appear coincidently with it. The senses are dulled and enfeebled, or intensified in certain directions, and impulses of any character may appear without premonition, like a flash of light, and disappear the next moment.

Every physician should be able to recognize these states, and study their meaning, both medico-legally and physiologically. In all probability medical interference in cases of supposed trance, in the form of an emetic or cathartic, or in the shape of a counter-irritant, would restore the normal recognition of environment, and save the patient untold suffering. The subject of inebriety must be studied above the dogmas of theologians and reformers, from the standpoint of science. It is purely a medical subject, and theories based on any other study are always "confusion worse confounded."—*Med. and Surg. Rep.*

---

A FRENCH photographer has invented an instrument for taking instantaneous negatives of birds and insects on the wing. It is an instrument somewhat resembling a gun, which is pointed at the flying creature.—*Ex.*

